# Integrated Proteomics and Metabolomics Reveal Spermine Enhances Sperm Freezability via Antioxidant Pathways

**DOI:** 10.3390/antiox14070861

**Published:** 2025-07-14

**Authors:** Lewei Guo, Zhuoxuan Gu, Bing Wang, Yunuo Wang, Jiaorong Chen, Yitong Li, Qiuju Zheng, Jing Zhao, He Ding, Hongyu Liu, Yi Fang, Jun Wang, Wenfa Lyu

**Affiliations:** 1 Key Laboratory of Animal Production, Product Quality and Security, Ministry of Education, Jilin Agricultural University, Changchun 130118, China; 20201575@mails.jlau.edu.cn (L.G.); 20220016@mails.jlau.edu.cn (Z.G.); wangbing111@jil.picc.com.cn (B.W.); 20230003@mails.jlau.edu.cn (Y.W.); cjr@mails.jlau.edu.cn (J.C.); liyichong@mails.jlau.edu.cn (Y.L.); 20220020@mails.jlau.edu.cn (Q.Z.); zhaojing@jlau.edu.cn (J.Z.); dinghe@jlau.edu.cn (H.D.); liuhongyu@jlau.edu.cn (H.L.); fangyi@iga.ac.cn (Y.F.); 2 Key Laboratory of Utilization and Protection of Beef Cattle Germplasm Resources, Jilin Agricultural University, Changchun 130118, China; 3 College of Animal Science and Technology, Jilin Agricultural University, Changchun 130118, China

**Keywords:** sperm freezability, oxidative stress, spermine, proteomics, metabolomics

## Abstract

Sperm freezability exhibits marked individual variability, yet the mechanisms remain unclear. Using bulls as the experimental model, we integrated proteomic (sperm) and metabolomic (seminal plasma) analyses of high-freezability (HF) and control (CF) bulls to identify key biomarkers associated with sperm freezability. Post-thaw motility and membrane integrity were significantly higher in HF bulls (*p* < 0.05). Sperm proteome analysis revealed upregulated antioxidant proteins (PRDX2, GSTM4), heat shock proteins (HSP70, HSP90), and key enzymes in arginine and proline metabolism (PRODH, LAP3). Seminal plasma metabolomics revealed elevated spermine in HF bulls. Meanwhile, we found that spermine abundance was positively correlated with post-thaw motility, as well as with the expression levels of both PRODH and LAP3 (r > 0.6, *p* < 0.05). Functional validation demonstrated that 200 μM spermine supplementation in cryopreservation extenders enhanced post-thaw motility, kinematic parameters (VAP, VSL, VCL), membrane integrity, and acrosome integrity (*p* < 0.05). Concurrently, spermine enhanced antioxidant enzyme (SOD, CAT, GSH-Px) activity and reduced ROS and MDA levels (*p* < 0.05). Our study reveals a spermine-driven antioxidant network coordinating sperm–seminal plasma synergy during cryopreservation, offering novel strategies for semen freezing optimization.

## 1. Introduction

Sperm cryopreservation is a vital technique in preserving male fertility and is widely employed in assisted reproduction, with the frozen semen quality becoming a critical indicator when evaluating male fertility potential [1,2]. Bulls are pivotal in advancing herd genetic progress. The primary determinants of semen cryopreservation success include the cryopreservation protocol and the bull’s genetic background [3,4]. Interindividual differences in sperm freezability are markedly observed in bovine herds [5,6,7]. Under identical freezing conditions, sperm from certain individuals tolerates cryopreservation-induced ROS and chromatin damage, while that from others exhibits significantly reduced post-thaw motility and membrane integrity [8]. This variation is governed by genetic factors [9], but the mechanistic basis of such heterogeneity remains poorly characterized.

The molecular mechanisms underlying cryodamage are complex and multidimensional. Excessive ROS accumulation induces lipid peroxidation (evidenced by elevated malondialdehyde, MDA), which compromises plasma membrane fluidity and mitochondrial function [10]. ROS simultaneously impair enzymatic antioxidant defenses, including glutathione (GSH), superoxide dismutase (SOD), and catalase (CAT) [11]. Furthermore, the ROS-mediated oxidation of DNA bases induces chromatin fragmentation, thereby undermining the early embryonic developmental potential [12]. Additionally, mechanical stress from ice crystal formation during freezing disrupts acrosomal integrity and flagellar microtubule systems, further diminishing sperm motility [13,14]. The cumulative effects of these injuries underscore the complexity of the regulatory networks governing sperm freezability.

Semen is primarily composed of spermatozoa and seminal plasma, which synergistically regulate freezability. Seminal plasma not only provides a nutrient-rich microenvironment for sperm but also delivers metabolites (antioxidants) that directly mitigate cryodamage [15]. In recent years, high-throughput sequencing technologies have emerged as pivotal tools in identifying biomarkers of sperm freezability [16,17]. Proteome analyses have identified critical markers associated with sperm freezability, including antioxidant proteins, namely peroxiredoxin-5 (PRDX5) [18] and glutathione S-transferase Mu 5 (GSTM5) [19]; molecular chaperones, namely heat shock protein 70 (HSP70) [20] and HSP90 [21]; and membrane-associated proteins, namely binder of sperm proteins (BSPs) [18,22] and voltage-dependent anion-selective channel 2 (VDAC2) [19]. Those discovered by metabolomics include acetyl-CoA and myo-inositol, which preserve sperm motility and function during cryopreservation [23]; amino acids, namely glutamate, alanine, and glycine, which reduce ice crystal formation via osmoregulation and suppress lipid peroxidation [24,25]; and unsaturated fatty acids, namely branched-chain fatty acids (BCFAs), oleic acid, and linoleic acid, which protect sperm plasma membrane integrity and alleviate freeze–thaw injury [26]. Moreover, seminal plasma polyamines, particularly spermine, have been identified as critical regulators of oxidative stress during sperm cryopreservation [9]. Spermine could scavenge ROS, stabilize DNA, and maintain membrane integrity by binding phospholipids, thus mitigating cryodamage [27,28], indicating its possible role in sperm freezability regulation. Sperm cryopreservation involves intricate regulatory mechanisms that cannot be fully elucidated by single-omics approaches alone. The KEGG pathway database serves as a critical tool for biomarker discovery in multi-omics studies, as it systematically bridges molecular signatures (differentially expressed proteins (DEPs) and differentially expressed metabolites (DEMs)) to higher-order biological functions [29,30]. By integrating KEGG enrichment with protein–metabolite interaction analysis, we can uncover the context-specific mechanisms underlying cryopreservation-induced cellular stress, which single-omics approaches often fail to capture.

**Figure 1:**
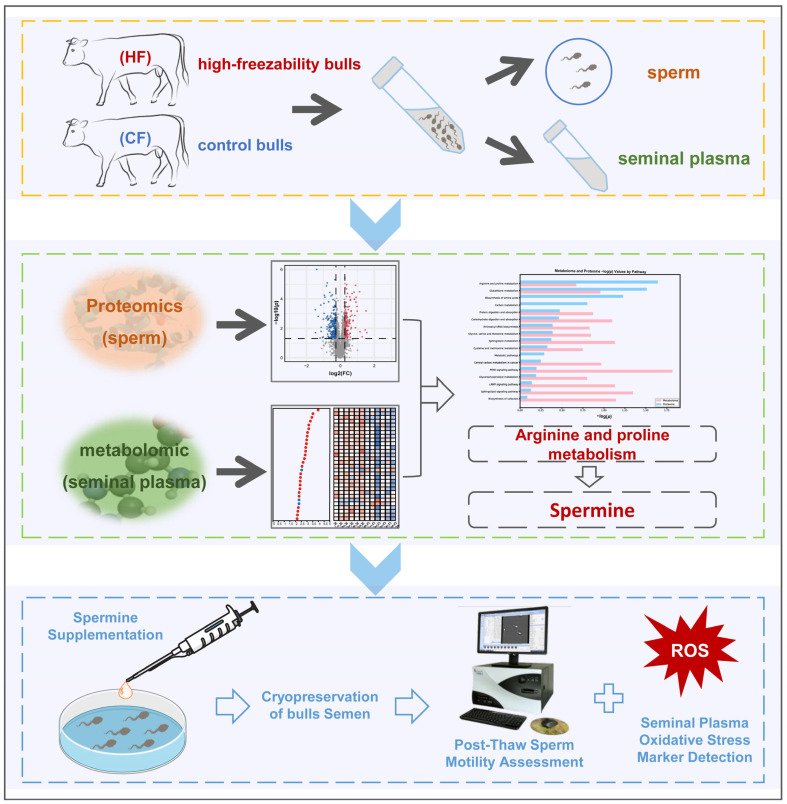
Experimental workflow diagram. Our experimental design involved collecting semen samples from high-freezability (HF) and control (CF) bulls, followed by proteomics of sperm and metabolomics of seminal plasma. The multi-omics data integration was performed through KEGG pathway enrichment and correlation analysis based on differentially expressed proteins (DEPs) and differentially expressed metabolites (DEMs) revealed key synergistic metabolic pathways, particularly arginine and proline metabolism. The cryoprotective role of spermine was further confirmed through functional validation experiments involving exogenous supplementation.

We integrated proteomic and metabolomic datasets from high-freezability (HF) and control (CF) bulls, screened coordinated pathways (including arginine and proline metabolism), and functionally validated spermine’s cryoprotective effects via exogenous supplementation (Figure 1). This study elucidates the molecular basis of the sperm–seminal plasma synergy in cryotolerance, providing a foundation for the optimization of bull selection and cryoprotectant design.

## 2. Materials and Methods

### 2.1. Experimental Animals and Ethical Issues

Simmental bulls were sourced from a bull station in Jilin, China. The use of these experimental bulls was approved by the Institutional Review Board at Jilin Agricultural University and adhered to all relevant ethical regulations.

### 2.2. Collection and Handling of Bull Semen

All breeding bulls were raised under standardized farm management with optimal feeding conditions and a confirmed good health status, certified for frozen semen production. Semen was collected by trained technicians using the artificial vagina method and immediately transported to the laboratory. The ejaculate was diluted with Optidyl extender (IMV Technologies, L’Aigle, France) to a final concentration of 96 × 10^6^ sperm/mL for fresh semen evaluation and cryopreservation.

For cryopreservation, diluted semen was loaded into 0.25 mL straws using an automated filling/sealing system. The straws were equilibrated at 4 °C for 3.5 h in a cooling chamber and then frozen in a programmable cryochamber with rapid cooling to −140 °C before storage in liquid nitrogen tanks.

### 2.3. Computer-Assisted Sperm Analysis (CASA)

CASA was performed using the IVOS II system (IMV Technologies, L’Aigle, France) to evaluate sperm motility and kinematic parameters in both fresh and thawed semen samples. All analyses were conducted by the same technician to ensure consistency. Fresh semen analysis included motility (%), progressive motility (%), and kinematic parameters (straight-line velocity (VSL, μm/s), curvilinear velocity (VCL, μm/s), distance straight-line (DSL, μm), distance curvilinear (DCL, μm), average path velocity (VAP, μm/s), distance average path (DAP, μm), amplitude of lateral head displacement (ALH, μm), beat-cross frequency (BCF, Hz), straightness (STR, %), linearity (LIN, %), and wobble (WOB, %)). For post-thaw samples, straws were thawed in a 37 °C water bath for 2 min before identical parameter assessments.

### 2.4. Sperm Plasma Membrane Integrity

**Figure 2:**
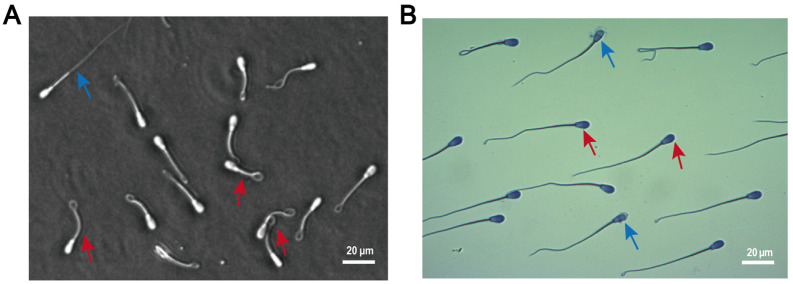
Morphological assessment of spermatozoa. (**A**) Plasma membrane integrity evaluated by HOST. Red arrows indicate spermatozoa with intact plasma membranes; blue arrows denote dead spermatozoa. (**B**) Acrosomal integrity assessed by Coomassie Brilliant Blue staining. Red arrows indicate intact acrosomes; blue arrows highlight damaged acrosomes.

The integrity of plasma membranes in both fresh and thawed spermatozoa was assessed using the hypo-osmotic swelling test (HOST). The experimental procedure was conducted as follows: semen samples were gently mixed with hypo-osmotic solution (1.351 g fructose + 0.735 g sodium citrate in 100 mL distilled water) by pipetting, followed by incubation at 37 °C for 30 min. Microscopic examination revealed that spermatozoa with intact plasma membranes exhibited characteristic coiled or curved tails due to osmotic swelling, while those with damaged membranes remained straight (Figure 2A). For each sample, a minimum of 200 spermatozoa were systematically evaluated and counted, with the numbers of spermatozoa displaying intact versus damaged plasma membranes recorded separately.

### 2.5. Sperm Acrosome Integrity

The acrosome integrity of both fresh and cryopreserved–thawed spermatozoa was evaluated by Coomassie Brilliant Blue staining. Briefly, semen samples were centrifuged at 2000× *g* for 5 min, followed by removal of the supernatant. The sperm pellet was resuspended in 100 μL PBS, with this washing procedure repeated twice. Subsequently, 5 μL of sperm suspension was smeared onto clean slides and air-dried at room temperature. The air-dried slides were stained with Coomassie Brilliant Blue solution in a staining jar for 30 min, followed by gentle rinsing with distilled water to remove excess dye. After air-drying completely, the slides were examined under a light microscope. Spermatozoa with intact acrosomes exhibited smooth and uniformly blue-stained apical ridges (Figure 2B), while those with damaged acrosomes showed partial or a complete absence of staining. A minimum of 200 spermatozoa were evaluated per sample to determine the percentage of acrosome-intact sperm.

### 2.6. Proteomic Sequencing Analysis of Spermatozoa

Based on the post-thaw sperm motility, 14 bulls were classified into high-freezability (HF, *n* = 7) and control (CF, *n* = 7) groups, with four randomly selected per group for proteomic analysis. Semen samples (200 µL per bull) were centrifuged at 1000× *g* for 5 min to remove seminal plasma, and sperm pellets were washed twice with PBS. The sperm proteome sequencing method strictly followed the experimental protocol provided by Lianchuan Biotech Co., Ltd. (Hangzhou, China). Washed sperm samples were lysed in protein lysis buffer using ice-bath sonication, followed by 30 min of incubation on ice. Lysates were cleared by centrifugation at 20,000× *g* for 10 min at 4 °C, and protein concentrations were determined using the BCA assay. For each sample, 100 µg of protein was reduced with DTT (30 °C, 60 min), alkylated to block free thiols, and digested overnight at 37 °C with trypsin. The resulting peptides were desalted using C18 SPE columns, vacuum-dried, and labeled with TMT reagents according to the manufacturer’s protocol. Labeled peptides were pooled and fractionated by basic-pH reverse-phase HPLC (C18 column). Fractions were collected based on UV detection, pooled, and dried. For LC-MS/MS analysis, fractions were reconstituted and separated on a nanoUPLC system (UltiMate 3000 nanoLC, Dionex, Sunnyvale, CA, USA) coupled to an Orbitrap mass spectrometer (Thermo Scientific, Waltham, MA, USA). Raw MS data were processed using MaxQuant (v1.5.2.8) against the UniProtKB/SwissProt protein database. The search parameters included false discovery rate (FDR) ≤ 1% at both the peptide and protein levels, precursor mass tolerance of 10 ppm, fragment ion mass tolerance of 0.02 Da, and a minimum requirement of one unique peptide per protein identification. The processed data were subsequently uploaded to the Lianchuan Cloud Platform for bioinformatic analysis.

DEPs were identified by *p* < 0.05 and fold change (FC) ≥1.2 or ≤0.83. The functional annotation of DEPs was performed through Gene Ontology (GO) enrichment analysis (biological process, molecular function, cellular component) and Kyoto Encyclopedia of Genes and Genomes (KEGG) pathway mapping to elucidate their roles in sperm cryotolerance regulation.

### 2.7. Non-Targeted Metabolomic Sequencing Analysis of Seminal Plasma

Six bulls were randomly selected from each of the HF and CF groups for metabolomic analysis. Semen samples (200 µL per bull) were centrifuged at 1000× *g* for 5 min to collect seminal plasma. Following Majorbio Biotech’s protocol, 100 μL seminal plasma was extracted with an acetonitrile–methanol solution (1:1 *v*/*v*) containing four internal standards (including 0.02 mg/mL L-2-chlorophenylalanine). The extraction involved vortex mixing for 30 s followed by cold ultrasonication at 5 °C and 40 kHz for 30 min. Samples were then precipitated at −20 °C for 30 min and centrifuged at 13,000× *g* and 4 °C for 15 min. The supernatant was nitrogen-dried and reconstituted in 100 μL of acetonitrile–water (1:1 *v*/*v*), followed by additional ultrasonication (5 °C, 40 kHz, 5 min) and final centrifugation (13,000× *g*, 4 °C, 10 min) before LC-MS/MS analysis. Quality control samples were prepared by pooling equal volumes from all samples and were inserted at every 5–10 experimental samples during the analytical run. The analysis was performed using a Thermo Fisher UHPLC-Orbitrap Exploris 240 system equipped with an HSS T3 column (100 mm × 2.1 mm inner diameter, 1.8 μm particle size). The mobile phase consisted of 0.1% formic acid in water–acetonitrile (95:5 *v*/*v*) as phase A and 0.1% formic acid in acetonitrile–isopropanol–water (47.5:47.5:5 *v*/*v*/*v*) as phase B, with a flow rate of 0.40 mL/min and the column temperature maintained at 40 °C. Mass spectrometry data were acquired in both positive and negative ion modes with a scan range of *m*/*z* 70–1050. Key MS parameters included spray voltages of 3500 V (positive) and −3000 V (negative), a sheath gas flow of 50 arbitrary units, an auxiliary gas flow of 13 arbitrary units, an ion source temperature of 450 °C, and cyclic collision energy of 20–40–60 V. The raw LC-MS data were processed with Progenesis QI for baseline correction, peak identification, retention time alignment, and the generation of a data matrix containing retention times, mass-to-charge ratios, and peak intensities. Metabolite identification was performed by matching the MS and MS/MS spectra against the HMDB and Metlin databases, along with Majorbio’s in-house library. The data matrix was preprocessed by applying the 80% rule for missing value filtering, sum normalization, and the removal of variables with QC sample relative standard deviations exceeding 30%, followed by log10 transformation. Multivariate statistical analysis, including principal component analysis and orthogonal partial least squares discriminant analysis, was conducted using the ropls package in R, with model stability assessed through 7-fold cross-validation. DEMs were identified based on the variable importance in projection (VIP) > 1 and *p* < 0.05. These metabolites were functionally annotated against the KEGG database to map enriched metabolic pathways, and topological analysis was subsequently conducted via MetaboAnalyst 5.0. All analyses were performed on the Majorbio Cloud Platform.

### 2.8. Bioinformatics Analysis

To systematically elucidate the synergistic regulatory mechanisms between the sperm proteome and seminal plasma metabolome in sperm cryotolerance, this study integrated multi-omics data and conducted the following analyses. We first performed KEGG enrichment analysis (KEGG Mapper, http://www.kegg.jp/kegg/mapper.html, accessed on 14 May 2025) on DEPs and DEMs to identify overlapping metabolic pathways. Subsequently, we conducted Pearson correlation analysis between DEPs/DEMs enriched in the overlapping pathways and post-thaw sperm motility to calculate correlation coefficients. Additionally, candidate biomarkers were prioritized based on their centrality in enriched pathways, experimental support from the cryopreservation-related literature, and significant correlations with post-thaw sperm motility (absolute correlation coefficient |r| ≥ 0.6 and *p* < 0.05) for the selection of sperm freezability candidate biomarkers.

### 2.9. Preparation and Handling of Semen Dilution Liquid

To investigate the effects of the cryoprotective marker spermine on the cryopreservation of bull semen, spermine was added to the freezing dilution liquid at concentrations of 0, 50 μM, 100 μM, 200 μM, and 400 μM. The bull semen was then frozen according to standard freezing protocols. Assessments were performed on frozen–thawed sperm motility, kinematic parameters, plasma membrane integrity, and acrosome integrity. The semen required for all experiments was obtained from three or more breeding bulls.

### 2.10. Enzyme-Linked Immunosorbent Assay (ELISA)

The oxidative stress levels (including SOD, CAT, and GSH-Px antioxidant enzyme activity and oxidative stress marker MDA and ROS concentrations) in post-thaw bull seminal plasma from semen samples supplemented with different spermine concentrations were measured using ELISA kits (MEIMIAN, Jiangsu Meimian Industrial Co., Ltd., Yancheng, China). Following the manufacturer’s protocol, briefly, thawed semen samples were centrifuged at 3000 rpm for 15 min to collect seminal plasma. For the assay, 10 µL of seminal plasma mixed with 40 µL of sample diluent buffer was added to each well and incubated at 37 °C for 30 min, after washing five times. Subsequently, 50 µL of horseradish peroxidase (HRP)-conjugated reagent was added, and the mixture was further incubated at 37 °C for 30 min, followed by five additional washes. Finally, the chromogenic substrate and stop solution were added sequentially, and the absorbance was measured at 450 nm using a microplate reader (BioTek, Winooski, VT, USA).

### 2.11. Statistical Analysis

The data are presented as the mean ± standard deviation (SD). Statistical analysis was performed using one-way ANOVA followed by Tukey’s honestly significant difference post hoc test. *p* < 0.05 was considered statistically significant. The statistical analyses were conducted using GraphPad Prism version 8.0 (San Diego, CA, USA).

## 3. Results

### 3.1. Measurement of Semen-Related Parameters in Bulls

**Figure 3:**
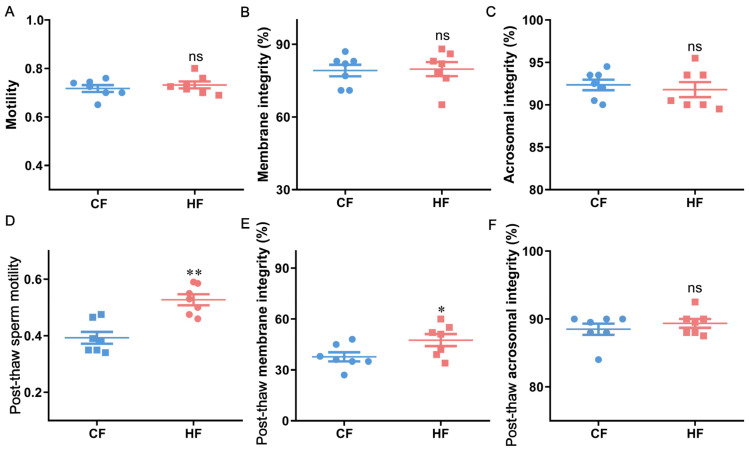
Sperm quality parameters in HF and CF bulls. Fresh sperm (**A**) motility, (**B**) membrane integrity, and (**C**) acrosome integrity; post-thaw sperm (**D**) motility, (**E**) membrane integrity, and (**F**) acrosome integrity. Data are presented as mean ± SEM (*n* = 7). Individual points represent HF and CF samples. ** *p* < 0.01, * *p* < 0.05, ns (not significant, *p* > 0.05).

The motility, plasma membrane integrity, and acrosome integrity of fresh sperm from bulls were assessed, along with the post-thaw motility, plasma membrane integrity, and acrosome integrity. Based on these parameters, 14 bulls were selected and divided into two groups: the HF group (*n* = 7) and CF group (*n* = 7). The results showed no significant differences (*p* > 0.05) in fresh sperm motility, plasma membrane integrity, or acrosome integrity between the two groups (Figure 3A–C). However, the post-thaw sperm motility and plasma membrane integrity in the HF group were significantly higher than those in the CF group (*p* < 0.05; Figure 3D,E), while no significant difference was observed in the post-thaw acrosome integrity (*p* > 0.05; Figure 3F).

### 3.2. Proteomic Analysis of Sperm Freezability

**Figure 4:**
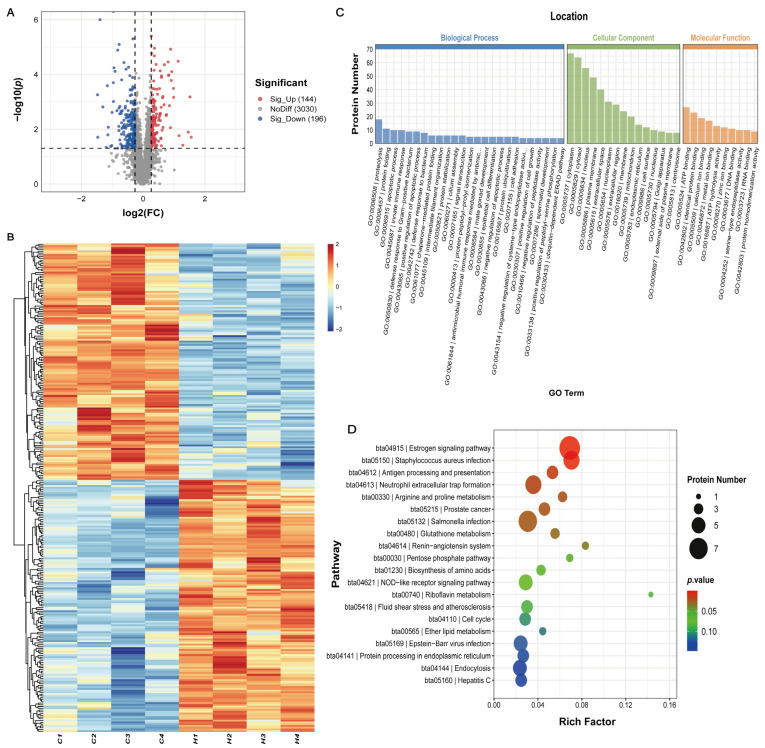
Identification and functional enrichment analysis of DEPs between HF and CF bulls. (**A**) Volcano plot of DEPs. The blue dots represent downregulated DEPs, red dots represent upregulated DEPs, and gray dots represent non-significant proteins. (**B**) Hierarchical clustering heatmap of DEPs. Red and blue indicate high and low expression levels, respectively. (**C**) Gene Ontology (GO) annotation of DEPs, classified into cellular components, biological processes, and molecular functions. The bar graph illustrates the distribution of DEP numbers across enriched terms in each category. For clarity, we present the top 25 biological processes, top 15 cellular components, and top 10 molecular functions, ranked by the number of annotated DEPs. (**D**) KEGG pathway enrichment analysis of DEPs. Bubble color indicates *p*-value significance, while size reflects gene count.

The proteomic analysis of sperm samples (n = 4) from the CF and HF groups identified 3370 proteins. As shown in Figure 4A, the volcano plot analysis categorized proteins into three types, with blue dots representing downregulated DEPs, red dots representing upregulated DEPs, and gray dots representing non-significant proteins. Compared to the CF group, the HF group exhibited 196 downregulated and 144 upregulated proteins, with a significance threshold set at FC ≥ 1.2 or ≤ 0.83 and *p* < 0.05. Hierarchical clustering analysis revealed distinct protein expression patterns between the groups (Figure 4B).

To further elucidate the biological functions of the DEPs, GO analysis was performed. Significantly enriched molecular function GO terms included ATP binding (e.g., WARS1, CKB, TKFC, PPP5C, VCP, and HSP70 family members HSPA1A, HSPA2, HSPA13, as well as HSP90 family member HSP90AB1), ATP hydrolysis activity (e.g., VCP, HSPA1A, HSPA2, HSPA13, HSP90AB1, and CCT family members CCT7/CCT8/CCT6A/CCT6B), calcium ion binding (e.g., CALR, RCN2, CALU, EHD4, CRELD2, GAS6), and metal ion binding (e.g., ME2, ARSK, TKTL1/TKTL2, TUBB5). Among biological processes, the DEPs were primarily enriched in apoptotic regulation (e.g., HSP90AB1, FLNA), cilium assembly (FLNA, EHD4, WDR19), and male gonad development (e.g., SFRP2). Cellular component analysis indicated that the DEPs were mainly localized in the cytoplasm, cytosol, nucleus, and plasma membrane (Figure 4C). KEGG pathway enrichment revealed that DEPs were primarily involved in the estrogen signaling pathway, arginine and proline metabolism, glutathione metabolism, and the renin–angiotensin system (Figure 4D).

### 3.3. Metabolomic Analysis of Seminal Plasma Freezability

**Figure 5:**
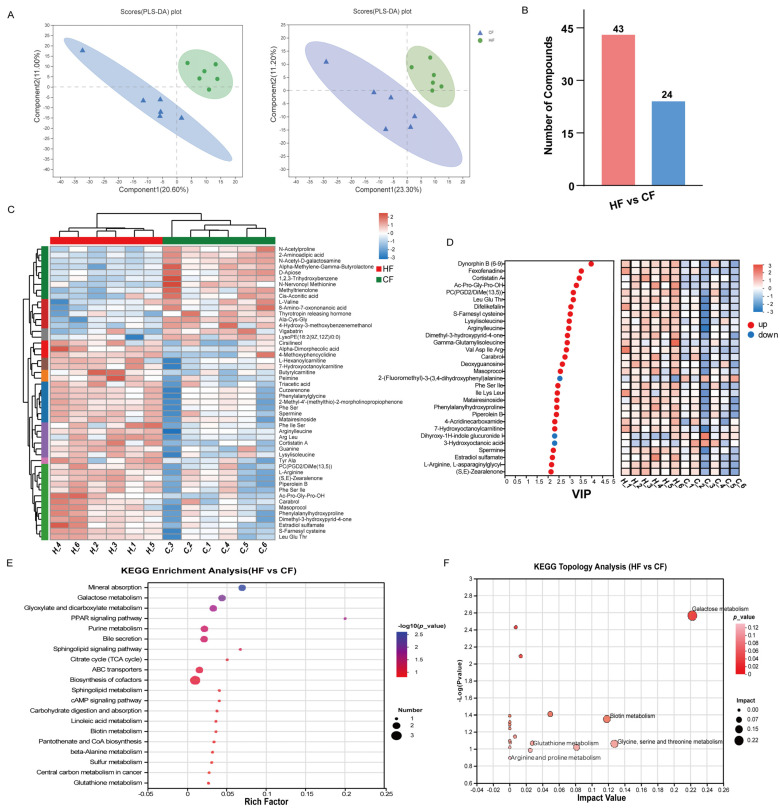
Metabolomic analysis of DEMs between HF and CF bulls. (**A**) PLS-DA score plots in positive (ESI+) and negative (ESI-) ionization modes. (**B**) Histogram showing counts of up- and downregulated DEMs between HF and CF groups. Pink bars represent up-regulated DEMs, and blue bars represent down-regulated DEMs. (**C**) Hierarchical clustering heatmap of DEMs between HF and CF groups. The color bar and numbers in the upper right corner represent the relative abundances of metabolites, with red indicating upregulated metabolites and blue indicating downregulated metabolites. (**D**) Top 30 DEMs ranked by VIP scores. The abscissa represents VIP values, while the ordinate shows the differential metabolites. Red and blue colors denote up- and downregulated metabolites, respectively. (**E**) KEGG pathway enrichment analysis of DEMs. The abscissa represents the rich factor and the ordinate displays the pathway names. Dot color indicates *p*-value (with red representing more significant enrichment), while dot size corresponds to number of metabolites involved in each pathway. (**F**) Pathway topological analysis plot. Dot color represents *p*-value and dot size indicates pathway impact.

The LC-MS analysis of seminal plasma (n = 6) from the CF and HF groups identified 1306 metabolites in positive ion mode and 940 in negative ion mode, totaling 2246 metabolites. PLS-DA analysis demonstrated separation between the CF and HF groups in both ion modes, indicating significant metabolic differences between these groups (Figure 5A). Through the comprehensive analysis of the metabolites in both ion modes using VIP > 1 and *p* < 0.05 as significance thresholds, we identified 67 DEMs, including 43 upregulated and 24 downregulated metabolites (Figure 5B). To visualize the expression patterns of these DEMs across groups, we generated a heatmap with hierarchical clustering analysis, where clustered DEMs exhibited similar expression profiles (Figure 5C). Figure 5D displays the top 30 DEMs with the highest VIP values, including spermine, Ac-Pro-Gly-Pro-OH, gamma-glutamylisoleucine, and 7-hydroxyoctanoylcarnitine.

To elucidate the biological significance of these DEMs, we performed KEGG pathway enrichment (*p* < 0.05) and topological analysis, which revealed their predominant involvement in the galactose metabolism; biotin metabolism; glycine, serine, and threonine metabolism; and glutathione metabolic pathways (Figure 5E,F). These findings suggest that these pathways may play critical roles in sperm cryopreservation processes.

### 3.4. Integrated Analysis of Sperm Proteome and Seminal Plasma Metabolome

**Figure 6:**
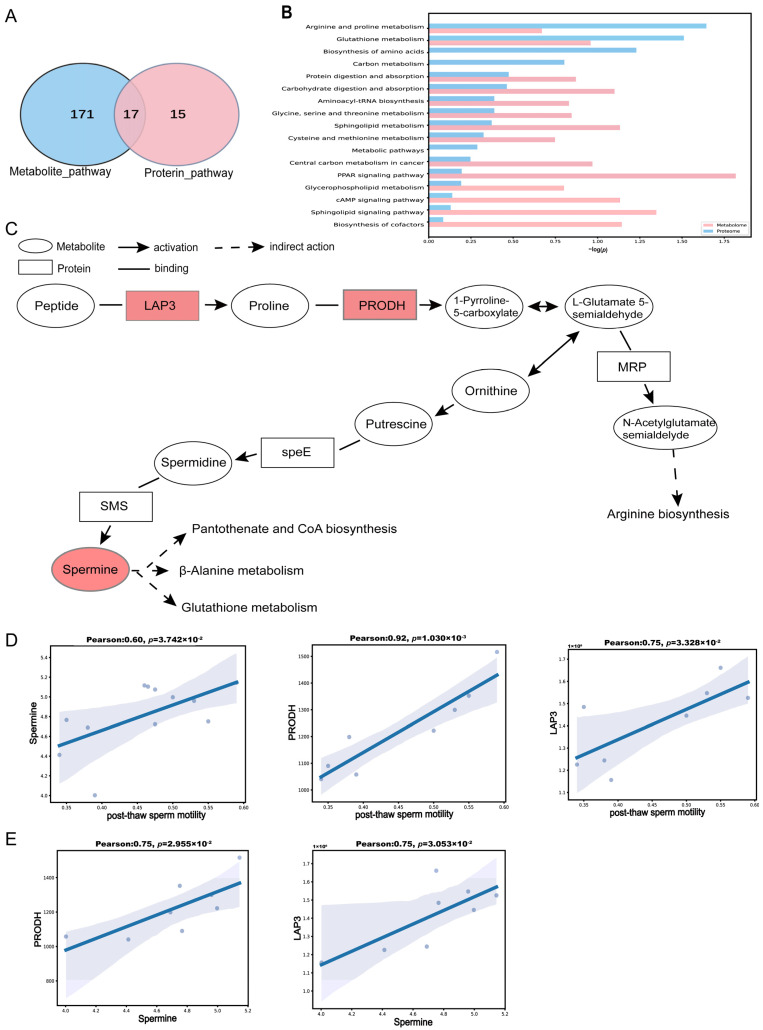
Integrated analysis of proteomic and metabolomic data. (**A**) Venn diagram showing the number of KEGG-enriched pathways for DEPs and DEMs, as well as the overlapping pathways. DEPs’ KEGG-enriched pathways are shown in blue; DEMs’ KEGG-enriched pathways are in pink. (**B**) Bar plot of co-enriched KEGG pathways for DEPs and DEMs. Blue bars represent proteins, and pink bars represent metabolites. (**C**) Network diagram of proteins and metabolites within the arginine and proline metabolism pathway. Circles represent metabolites, rectangles represent proteins, red circles indicate upregulated metabolites, red rectangles indicate upregulated proteins, white circles denote metabolites with no significant change in abundance, and white rectangles denote proteins with no significant change in expression. (**D**) Pearson correlation analysis between post-thaw sperm motility and spermine levels, PRODH expression, and LAP3 expression. (**E**) Pearson correlation analysis between spermine levels and expression of PRODH and LAP3.

To explore the relationships between seminal plasma DEMs and spermatozoa, we conducted an integrated analysis of the seminal plasma metabolomic and sperm proteomic data. As shown in the Venn diagram (Figure 6A), a comparison of the KEGG enrichment results between the DEPs and DEMs revealed significant overlap in 17 pathways, including arginine and proline metabolism, glutathione metabolism, and the PPAR signaling pathway (Figure 6B). In Figure 6C, using arginine and proline metabolism as an example, it can be seen that the HF group showed significantly upregulated protein expression of PRODH and LAP3, accompanied by increased accumulation of spermine (*p* < 0.05). To further investigate the association between these molecules and post-thaw sperm motility, we performed a Pearson correlation analysis. The results demonstrated that the PRODH, LAP3, and spermine levels were all positively correlated with post-thaw sperm motility (r > 0.6, *p* < 0.05; Figure 6D). Meanwhile, positive correlations were also observed between PRODH/LAP3 protein expression and the spermine concentration (r > 0.7, *p* < 0.05; Figure 6E). These findings suggest that PRODH and LAP3 may enhance sperm freezability by driving arginine and proline metabolism and spermine synthesis.

### 3.5. Effects of Spermine on Post-Thaw Sperm Kinematics and Morphology

**Figure 7:**
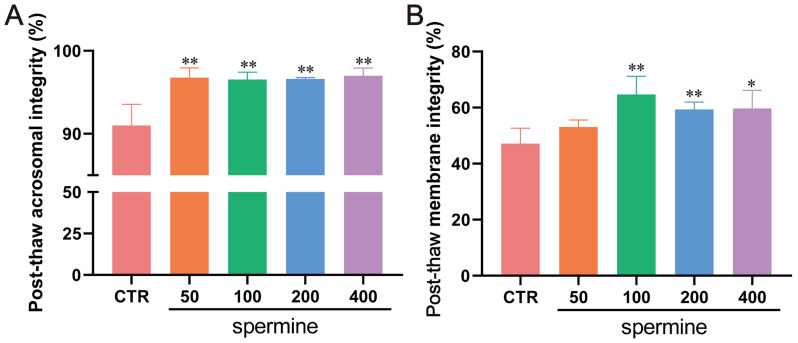
Effects of spermine supplementation on post-thaw sperm quality in bulls. (**A**) Acrosome integrity and (**B**) plasma membrane integrity of post-thaw sperm treated with varying concentrations of spermine. Data represent mean ± SD. ** *p* < 0.01, * *p* < 0.05 vs. CTR.

To validate spermine’s cryoprotective role, varying concentrations (0, 50, 100, 200, 400 μM) were added to freezing extenders. We found that spermine supplementation significantly improved the post-thaw sperm motility and kinematic parameters, including the distance average path (DAP), average path velocity (VAP), straight-line velocity (VSL), and curvilinear velocity (VCL) (*p* < 0.05; Table 1). However, no significant differences were observed in linearity (LIN), the beat-cross frequency (BCF), or the wobble coefficient (WOB) (*p* > 0.05). Morphological analysis confirmed the enhanced acrosome integrity (Figure 7A) and plasma membrane integrity (Figure 7B) in the spermine-treated groups.

### 3.6. Spermine Reduces Oxidative Stress in Bull Sperm

**Figure 8:**
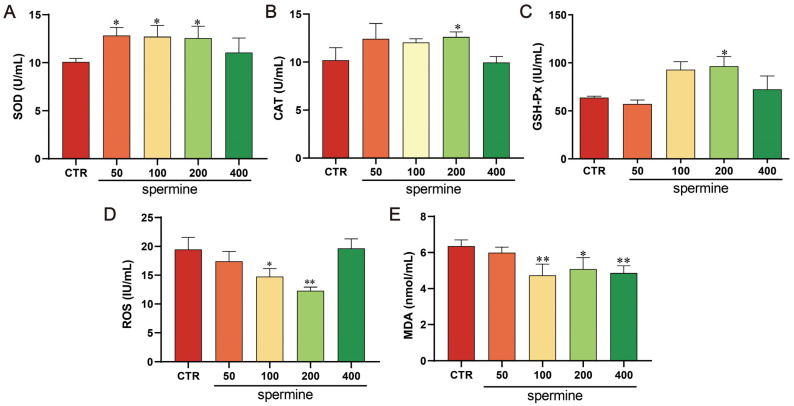
Effects of spermine supplementation in freezing medium on post-thaw bull sperm oxidative status. (**A**) Superoxide dismutase (SOD), (**B**) catalase (CAT), and (**C**) glutathione peroxidase (GSH-Px) activity; (**D**) reactive oxygen species (ROS) levels; and (**E**) malondialdehyde (MDA) levels. Data are presented as mean ± SD. ** *p* < 0.01, * *p* < 0.05 vs. CTR.

Supplementation with 200 μM spermine significantly increased the seminal plasma SOD, CAT, and GSH-Px activity (*p* < 0.05; Figure 8A–C) while reducing the MDA and ROS levels (*p* < 0.05; Figure 8D,E), demonstrating its role in alleviating oxidative damage during cryopreservation.

## 4. Discussion

Cryopreservation critically impacts sperm’s fertilizing capacity. The freeze–thaw process induces multiple sperm injuries, cryocapacitation (premature capacitation) [31], and oxidative stress, significantly impairing sperm motility and membrane integrity, thereby severely reducing AI success rates [32]. Sperm cryotolerance serves not only as a key biomarker in assessing the organismal health status but also effectively reflects the overall adaptive capacity of organisms regarding environmental changes, oxidative stress, and exogenous factors (e.g., pollutant exposure) [33]. Bulls with higher cryotolerance exhibit superior post-thaw motility, lower oxidative stress levels, and improved AI conception rates [34]. However, metabolic and proteomic biomarkers predictive of bovine sperm cryotolerance remain poorly characterized. This study evaluated sperm motility and morphological features and conducted integrated proteomic and seminal plasma metabolomic analyses to identify potential biomarkers linked to cryodamage, providing novel insights into the biological mechanisms underlying bovine sperm freezability.

Post-thaw sperm motility is a key phenotypic indicator when evaluating sperm cryotolerance, directly affecting sperm’s ability to migrate through the female reproductive tract and fuse with the oocyte. This process is regulated by both extracellular and intracellular factors [35]. While the fresh sperm quality did not differ between HF and CF bulls, marked divergence in post-thaw motility and membrane integrity emerged, indicating that cryotolerance relies on molecular responses to cryostress. Oxidative stress is a key driver of cryodamage. During cryopreservation, excessive ROS accumulation leads to ice crystal formation and sperm damage [36], necessitating a dynamic balance between ROS production and clearance [37]. Proteomic profiling revealed the significant upregulation of anti-apoptotic proteins (HSP90AB1, FLNA), antioxidants (PRDX2, GSTM4), and energy metabolism-related proteins (ATP-dependent chaperones HSPA1A, HSPA2, CCT family members CCT7/CCT8/CCT6A/CCT6B) in HF bulls. These findings align with prior studies showing that heat shock protein (HSP70, HSP90) expression levels correlate positively with post-thaw sperm quality [2,38,39], suggesting that HF bulls may stabilize protein conformation via upregulated HSPA1A, HSPA2, and HSP90AB1 to maintain sperm motility. PRDXs and GSTM belong to the antioxidant protein family; PRDX2 significantly reduces the intracellular ROS and MDA levels, enhancing post-thaw sperm motility and viability [40]. The enrichment of PRDX2 and GSTM4 in the HF group further confirms that oxidative stress regulation is a critical mechanism of cryotolerance. Collectively, these results indicate that HF bulls mitigate cryodamage and preserve post-thaw motility by reducing oxidative stress and maintaining heat shock protein expression.

Arginine promotes motility via nitric oxide synthesis [41], while proline acts as an osmoprotectant to alleviate cryodamage [42,43]. In this study, the activation of key enzymes in arginine–proline metabolism, namely PRODH and LAP3, enhanced cryotolerance by promoting polyamine (spermine) synthesis and strengthening the antioxidant defenses. This aligns with Yang et al. [44], who demonstrated this pathway’s role in regulating oxidative stress and apoptosis, underscoring its importance in sperm freezability. Metabolomics is particularly suitable for studying the effects of environmental factors on organisms, as it enables the analysis of complex interactions between metabolic pathways and metabolites, especially those related to oxidative stress metabolism [45]. Metabolomic analysis revealed the significant enrichment of lipids, organic acids, and derivatives, especially spermine accumulation, in the HF group. The positive correlation between spermine and post-thaw motility (r > 0.6, *p* < 0.05), coupled with its stimulation of antioxidant enzymes (SOD, CAT, GSH-Px), suggests ROS scavenging as a mechanism to reduce lipid peroxidation (lower MDA levels) and membrane damage. The co-enrichment of glutathione metabolism (GSTM4, LAP3) and spermine-driven polyamine metabolism further highlights oxidative stress mitigation as central to cryotolerance. These findings align with seminal plasma’s role as a sperm microenvironment modulator [46], indicating synergistic regulation between seminal metabolites and sperm proteomes.

This study presents the pioneering experimental validation of spermine’s cryoprotective effects in bovine sperm. Supplementation with 200 μM spermine significantly improved the post-thaw motility, kinematic parameters (VAP, VSL, VCL), acrosome integrity, and plasma membrane integrity. Mechanistically, spermine likely operates via direct ROS scavenging to reduce oxidative damage and the activation of PRODH-mediated proline metabolism, enhancing the endogenous antioxidant capacity through polyamine synthesis. This result aligns with the function of spermine in maintaining membrane stability and DNA integrity in sperm after freeze–thaw in pigs, horses, and dogs, indicating the conservation of spermine across different species [27,28,47]. This study has several limitations. Although our proteomic and metabolomic analyses identified arginine and proline metabolism as a potential key pathway in sperm cryotolerance and established correlations between PRODH/LAP3 and spermine levels, the precise mechanistic relationships require further experimental validation and detailed analysis. Additionally, our verification was limited to exogenous spermine supplementation; we did not conduct in vivo animal experiments, such as systemic administration via injection or dietary supplementation, to validate whether spermine could effectively improve semen cryoresistance.

## 5. Conclusions

This study identifies spermine as a key cryoprotectant in bovine semen. Integrated proteomics and metabolomics revealed that spermine accumulation in high-freezability bulls correlates with enhanced arginine and proline metabolism (PRODH, LAP3) and antioxidant defenses (GSTM4, LAP3). Supplementing extenders with 200 μM spermine significantly improved the post-thaw motility, acrosome integrity, and membrane integrity (*p* < 0.05), reducing ROS and MDA and activating SOD, CAT, and GSH-Px, offering a novel strategy for semen cryopreservation.

## Figures and Tables

**Table 1:** Effects of spermine on post-thaw bull sperm motility and kinematic parameters.

	CTR	50 μM	100 μM	200 μM	400 μM
Sperm viability (%)	62.28 ± 3.89 ^b^	73.35 ± 5.35 ^a^	76.87 ± 1.41 ^a^	70.93 ± 2.57 ^a^	61.82 ± 5.01 ^b^
Sperm motility (%)	31.90 ± 2.75 ^b^	45.25 ± 6.97 ^a^	48.13 ± 0.99 ^a^	43.67 ± 3.62 ^a^	43.82 ± 8.28 ^a^
DAP (μm)	27.21 ± 1.30 ^b^	33.16 ± 1.51 ^a^	34.58 ± 1.83 ^a^	29.43 ± 1.60 ^b^	33.55 ± 1.82 ^a^
DSL (μm)	21.54 ± 1.35 ^b^	26.62 ± 1.41 ^a^	28.52 ± 1.52 ^a^	23.93 ± 1.36 ^b^	28.05 ± 1.61 ^a^
DCL (μm)	47.07 ± 1.73 ^c^	56.36 ± 2.93 ^a^	58.92 ± 3.32 ^a^	51.16 ± 2.73 ^bc^	53.88 ± 2.32 ^ab^
VAP (μm/s)	73.03 ± 3.44 ^c^	91.08 ± 4.11 ^ab^	96.61 ± 3.74 ^a^	84.92 ± 2.97 ^b^	88.03 ± 3.27 ^b^
VSL (μm/s)	58.21 ± 2.82 ^c^	73.65 ± 4.27 ^ab^	79.52 ± 3.25 ^a^	69.24 ± 3.54 ^b^	74.53 ± 2.79 ^ab^
VCL (μm/s)	124.19 ± 4.97 ^d^	152.20 ± 6.67 ^ab^	162.59 ± 6.86 ^a^	143.52 ± 4.84 ^bc^	137.55 ± 5.87 ^c^
STR (%)	79.82 ± 1.19 ^b^	81.05 ± 1.38 ^ab^	82.72 ± 1.35 ^a^	81.48 ± 1.64 ^ab^	82.52 ± 1.47 ^ab^
LIN (%)	48.98 ± 1.40	50.53 ± 1.38	51.42 ± 1.27	51.25 ± 1.15	51.68 ± 1.28
ALH (μm)	6.37 ± 0.21 ^b^	7.20 ± 0.25 ^a^	7.37 ± 0.26 ^a^	6.82 ± 0.23 ^ab^	6.57 ± 0.36 ^b^
BCF (Hz)	24.76 ± 0.62 ^abc^	24.34 ± 0.71 ^b^	26.19 ± 0.53 ^a^	25.29 ± 5.73 ^ab^	23.91 ± 0.83 ^c^
WOB (%)	60.06 ± 1.12	61.29 ± 0.98	60.94 ± 0.71	60.23 ± 1.04	64.07 ± 0.43

Values are expressed as mean ± SD. Different lowercase letters within a row indicate statistically significant differences (*p* < 0.05). DAP, distance average path; DSL, distance straight-line; DCL, distance curvilinear; VAP, average path velocity; VSL, straight-line velocity; VCL, curvilinear velocity; STR, straightness (VSL/VAP); LIN, linearity (VSL/VCL); ALH, amplitude of lateral head displacement; BCF, beat-cross frequency; WOB, wobble coefficient (VAP/VCL).

## Data Availability

The raw data supporting the conclusions of this article will be made available by the authors upon request.
